# Effects of Repetitive Transcranial Magnetic Stimulation at the Cerebellum on Working Memory

**DOI:** 10.3390/brainsci13081158

**Published:** 2023-08-03

**Authors:** Jiangnan Yao, Bo Song, Jingping Shi, Kuiying Yin, Wentao Du

**Affiliations:** 1 Nanjing Research Institute of Electronic Technology, Nanjing 210019, China; 2 Department of Neurology, The Affiliated Brain Hospital of Nanjing Medical University, Nanjing 210029, China

**Keywords:** repeated transcranial magnetic stimulation (rTMS), cerebellum, working memory (WM), event-related potentials (ERP), brain network

## Abstract

Transcranial magnetic stimulation is a widely used brain intervention technique in clinical settings. In recent years, the role of the cerebellum in learning and memory has become one of the hotspots in the field of cognitive neuroscience. In this study, we recruited 36 healthy college or graduate students as subjects and divided them into groups, with 10 to 14 subjects in each group. We performed 5 Hz and 20 Hz repeated transcranial magnetic stimulation and sham stimulation on the Crus II subregion of the cerebellum in different groups, then let them complete the 2-back working memory task before and after the stimulation. We simultaneously recorded the electroencephalogram in the experiment and analyzed the data. We found that after repeated transcranial magnetic stimulation of the cerebellum at 5 Hz and 20 Hz, the N170 and P300 event-related potential components in the prefrontal cortex showed significant differences compared to those in the sham stimulation group. Using phase-locked values to construct brain networks and conduct further analysis, we discovered that stimulation frequencies of 5 Hz and 20 Hz had significant effects on the local and global efficiency of brain networks in comparison to the sham stimulation group. The results showed that repeated transcranial magnetic stimulation on cerebellar targets can effectively affect the subjects’ working memory tasks. Repeated transcranial magnetic stimulation at 5 Hz and 20 Hz could enhance the excitatory responses of the frontal lobes. After stimulation at 5 Hz and 20 Hz, the efficiency of the brain network significantly improved.

## 1. Introduction

Working memory (WM) is a cognitive ability that involves the temporary retention and utilization of information in the mind [1]. WM is extensively involved in the execution of complex tasks such as learning, decision-making, and reasoning. Crucially, the decline in WM is a major factor in the cognitive impairments that accompany aging. Therefore, different stimulation methods are currently being explored as potential interventions to enhance these capabilities, including non-invasive stimulation methods such as transcranial magnetic stimulation (TMS) and ultrasound stimulation. It also includes invasive stimulation such as deep brain electrical stimulation, which usually requires specialized surgical assistance. The two types of stimulation are used in different occasions, and the level of intensity varies. In preventive intervention, invasive stimulation is generally not acceptable due to the level of difficulty and pain involved. On the other hand, non-invasive stimulation is less painful, carries a lower risk, and can be performed without surgery. Therefore, it is more commonly used in intervention treatments.

Transcranial magnetic stimulation (TMS) is a non-invasive intervention technique used to stimulate the human brain and investigate its physiological mechanisms. At present, TMS technology has been used in the treatment of Alzheimer’s disease, stroke, depression, and other neuropsychiatric diseases [2,3]. The brain regions selected for stimulation have a crucial influence on the effects of stimulation [4]. Target regions are typically selected based on the target function and the brain circuit mechanism of cognitive processing [4,5]. The target regions of TMS intervention in the past have mainly been in the cerebral hemisphere. The most commonly studied sites in research on cognition and memory include the left dorsolateral prefrontal cortex, the right inferior frontal gyrus, the right superior temporal gyrus, and the precuneus cortex [6,7]. The stimulation frequency affects the neuromodulation effect of TMS [8]. At present, repetitive transcranial magnetic stimulation (rTMS) is widely used in clinical practice. High-frequency (frequency > 1 Hz) rTMS can increase cortical excitability, induce long-term potentiation, and enhance synaptic plasticity. Low-frequency (frequency ≤ 1 Hz) rTMS can inhibit cortical excitability, reduce synaptic activity, and induce long-term depression. In general, the higher the frequency of rTMS, the stronger the excitatory effect. However, frequencies higher than 20 Hz are rarely used due to safety concerns and subject tolerance. In addition, different intensities of stimuli also have varying effects [9]. Studies have found that different brain wave oscillation frequencies are involved in various brain activities, and theta oscillation waves may serve as an indicator of memory and attention processing [10]. However, there is no uniform standard for the frequency of TMS stimulation used to improve cognitive function.

The role of the cerebellum in learning and memory has become a prominent topic in the field of cognitive neuroscience. The surface of the human cerebellar cortex is folded much more tightly than that of the cerebral cortex. Martin I et al. found that the surface area of the cerebellum is significantly larger than previously documented, accounting for approximately 78% of the total surface area of the human neocortex [11]. In previous studies, the conclusions about the cerebellum were mostly related to motor function [12], but did not establish a connection between the cerebellum and cognitive memory. However, recent studies have shown that the function of the cerebellum is not simply involved in the coordination of body movements, but it also plays a role in regulating cognitive activities such as working memory, cognitive control, and reward expectation through cerebrum-cerebellar circuits. Many functions of the cerebellum have been gradually explored. Studies have found that the possible regulatory mechanism of cerebellar involvement in cognitive function is through a wide range of neural circuits in the brain, particularly those in the frontoparietal temporal lobe and limbic system, which affect the brain’s higher-level cognitive processes [13,14,15,16,17]. In studies of cerebellar activation with n-back tasks in healthy adults, researchers found significant activation in the Crus I/II regions of the cerebellum. Furthermore, the activation in this lobe increased with higher task load [18]. A functional map of the cerebellum published in 2019 in the journal *Nature Neuroscience* revealed that different regions of the posterior lobe of the cerebellum are associated with various cognitive functions [19]. The Crus II region of the cerebellum is associated with episodic memory and semantic prediction, while the Crus I/II region of the cerebellum is involved in prefrontal cortex function and regulation of the default mode network [19].

TMS in the cerebellum has been shown to modulate the default mode network [20], the attention network [20,21], and the functional connectivity between the cerebellum and the frontal lobe and other regions [14]. It is known that the application of right lateral cerebellar continuous theta-burst stimulation (cTBS) can reduce the amplitude of motor evoked potential (MEP) [22,23,24,25,26,27,28], and lateral cerebellar cTBS has been used in the treatment of various nervous system diseases, such as improving the motor symptoms of dystonia [29,30] and essential tremor [31]. Currently, there are well-established studies and clinical applications of the regulation of cerebellar activity by theta-burst stimulation (TBS). However, there are few studies on the various modes of rTMS protocols, and research on cerebellar cognitive memory function is even more scarce. [32,33,34].

Our research group has previously utilized a deterministic fiber-tracking algorithm and graph theory analysis to discover that various cognitive functions are impaired after posterior cerebellar infarction. We found that both the global and local efficiency attributes of the brain network are decreased [35]. Specifically, the efficiency of key nodes such as the bilateral precuneus, frontotemporal lobe, and cingulate gyrus, which are associated with cognition, are significantly changed. The results show that the posterior cerebellum plays an important role in integrating and regulating the global and cognitive networks of the brain. After 4 weeks of 5 Hz rTMS intervention in the bilateral cerebellar Crus Ⅱ, Alzheimer Disease (AD) patients showed significant improvement in overall cognitive function, memory, attention, visuospatial ability, and executive function [36]. However, it is not clear which stimulation frequency of cerebellar intervention can most effectively improve cognitive levels. In this regard, intervention studies with different frequencies of cerebellar stimulation are needed.

Based on previous findings, this study aimed to determine the effects of rTMS at different frequencies on working memory. The objective was to identify the frequency that is most effective in enhancing working memory. At the current stage of research, we believe that it is necessary to collect not only experimental data from patients, but also certain data from individuals who are considered normal. Therefore, in this study, we selected normal subjects and performed rTMS stimulation on the Crus II subregion of their cerebellum. EEG was collected and analyzed during the 2-back task before and after the stimulation. We aim to investigate the effect of cerebellar rTMS at different frequencies on working memory function in healthy individuals. Our goal is to establish reference samples for the treatment of cognitive impairment-related diseases and explore the potential for pre-disease intervention to delay the onset of disease in susceptible individuals.

## 2. Materials and Methods

### 2.1. Design of Experiments

The aim of this study was to examine the effects of rTMS at different frequencies on EEG components related to working memory (WM) and brain network connectivity. The commonly used paradigms are “go-nogo” and “n-back.” The go-nogo paradigm primarily evaluates executive function, while the n-back paradigm primarily evaluates memory function [37,38]. The ERP generated by the n-back paradigm is relatively simple, while the go-nogo paradigm is more complex. In this experiment, the use of a simple 2-back paradigm can eliminate interference from executive function and allow for a focus on WM. In order to observe the effects of stimulation on EEG, a before and after comparison is essential. Therefore, it is important for the same subject to conduct two sets of experiments within a short time frame, rather than at longer intervals. This ensures that the experimental conditions and the subject’s state remain consistent. Additionally, to account for the learning effect, it is important to establish a control group in which subjects receive a sham stimulus. The results from this group can serve as a benchmark for comparison.

### 2.2. Screening of Subjects

The study was conducted according to the guidelines of the Declaration of Helsinki and approved by the Ethics Committee of the Affiliated Brain Hospital of Nanjing Medical University (2022-KY026-01, approved date: 24 February 2022).

Subject recruitment was conducted from January to June 2022, with plans to recruit younger, healthy subjects.

Inclusion criteria: 36 healthy college students and graduate students aged 18–30 years old were selected. Their Mini-Mental State Examination (MMSE) and Montreal Cognitive Assessment (MOCA) scores were within the normal range. Hamilton Anxiety (HAMA), Hamilton Depression (HAMD) < 7 points. They were right-handed (left hemisphere dominant). Magnetic resonance imaging (MRI) of the head was performed, and no organic disease was found.

Exclusion criteria: patients with a history of neurological diseases that affect cognitive function, such as brain trauma, stroke, epilepsy, and others; patients with a history of mental illness and a related family history or other factors; patients with severe primary diseases of the cardiovascular, hepatic, renal, and hematopoietic systems; patients with an implanted medical device; patients who are unable to cooperate in completing examinations or follow-up visits.

The specific conditions of the subjects are shown in Table 1, and the full names of the subjects have been concealed.

### 2.3. Process of Experiment

Before participating in the experiment, the subjects needed to understand the 2-back task in order to achieve a basic level of proficiency. We put the EEG cap on the subject correctly, set the acquisition device, and then let the subject perform the 2-back task approximately 30 times. This task required the subject to use a computer screen and keyboard. The screen was used to display the characters, while the keyboard was used for input. There are two buttons on the keyboard, J and F, which correspond to the judgments of right and wrong. When the subjects indicated that they understood the 2-back procedure, the experiment officially began. The experimenter then explained the complete procedure to the participants, excluding key factors such as stimulus grouping and the purpose of the experiment, before commencing the experiment.

We used an EEG cap with 69 + 2 channels (Neuracle Technology (Changzhou) Co., Ltd., Changzhou, Changzhou, China), and the EEG signal acquisition device is also from the same company [39].

**Figure 1:**
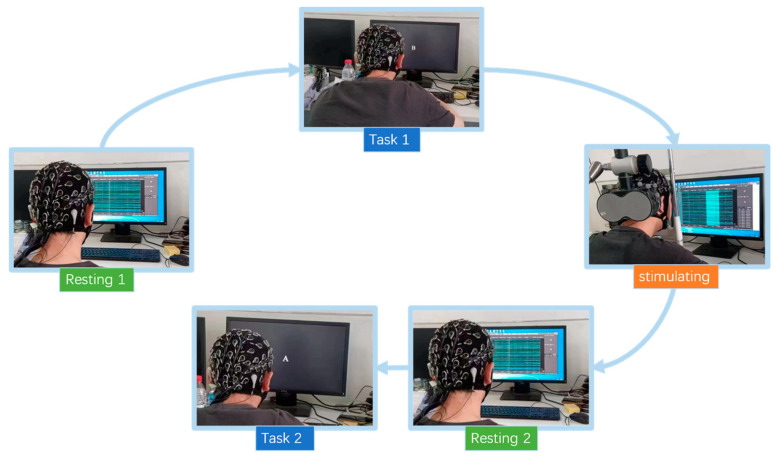
Flow chart of the experiment.

When the subjects formally started the 2-back task, each subject needed to complete two sets of experiments, as shown in Figure 1. In the four processes depicted in the figure, the EEG acquisition equipment is in the normal acquisition state, and it is synchronized with the 2-back experimental procedure to facilitate the segmentation of the experimental task.

**Figure 2:**
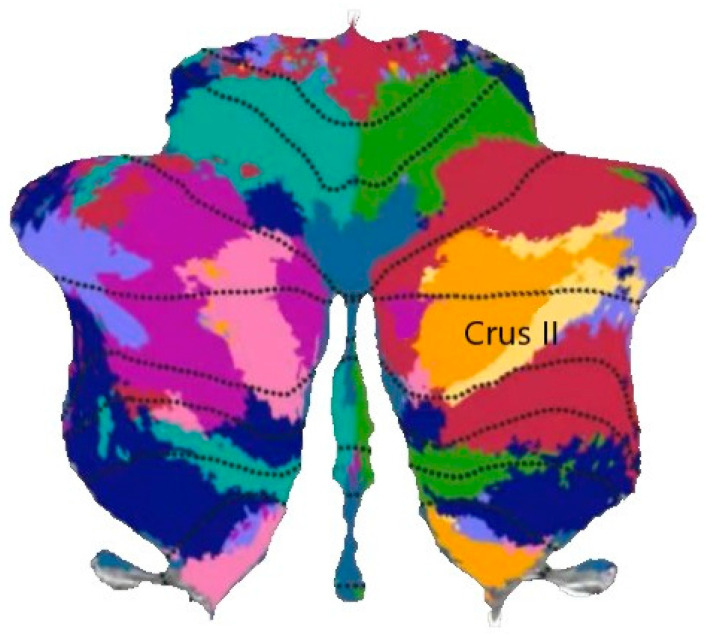
Use rTMS to stimulate Crus II of cerebellum.

During the stimulation phase, all subjects received rTMS at a consistent frequency, position, and power on the Crus II region of the cerebellum, as shown in Figure 2. The choice of position depended on the localization of MRI data. The stimulus intensity was 80% RMT. In the experiment, all the subjects exhibited a high tolerance to stimulation, and none of them experienced any adverse reactions such as headaches, dizziness, or nausea.

There were three types of stimuli: 5 Hz, 20 Hz, and sham. Each subject might receive one of the stimuli, but they would not learn which stimulus they received until after the experiment was complete. The stimulation methods were grouped randomly, without considering the age, education, or other factors of the subjects. No matter which group, a simple 2-back task was conducted. Due to its simplicity and lack of a threshold, it can ensure that the ERP components of each individual are roughly similar. This helps to partially mitigate the memory differences among the subjects.

**Figure 3:**
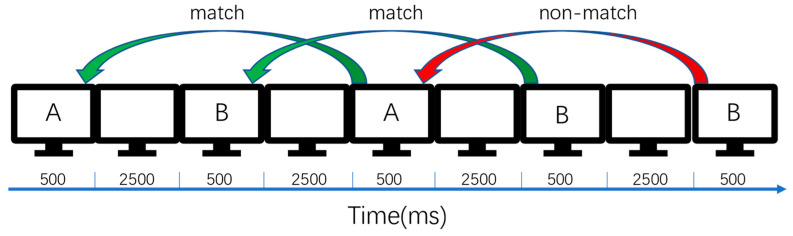
Flow of the 2-back task.

The 2-back task used random characters A and B. After the characters appeared on the screen, the subjects had two seconds to react and press a key, followed by one second of rest time. This process repeated for each event, resulting in a total of 148 events. If we include the number before and after the stimulus, the total number of events would be 296, as shown in Figure 3.

### 2.4. EEG Analysis Method

We used a general process for EEG analysis, based on the MATLAB and EEGLAB toolbox. The following steps were used for preprocessing.

**Figure 4:**
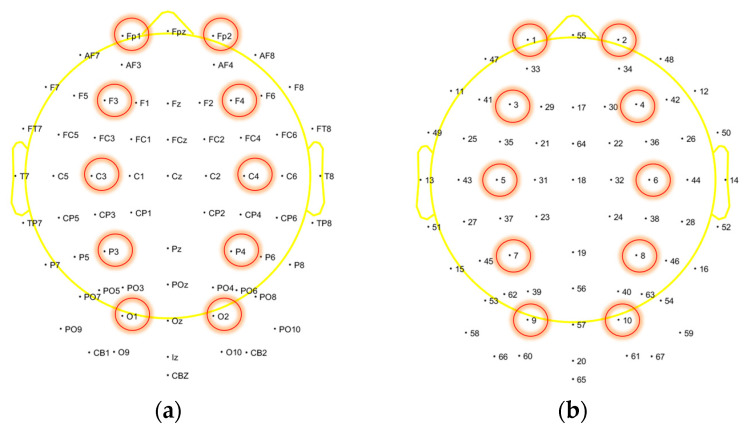
Channel location. The channels circled in the picture were used for ERP analysis. (**a**) Channels’ name; (**b**) Channels’ number.

First, channel location. Channels and their number are shown in Figure 4.

Second, we filtered the data twice. A 48 to 52 Hz band-stop filter was designed to filter out 50 Hz frequency wave interference in the power supply system, and a 0.5 to 80 Hz band-pass filter was designed to remove high-frequency non-EEG components.

Third, we segmented the entire data. All original time points were retained. Then, we removed bad channels, and they were interpolated in adjacent channels.

Fourth, we performed whole-brain mean weight reference and removed the baseline.

**Figure 5:**
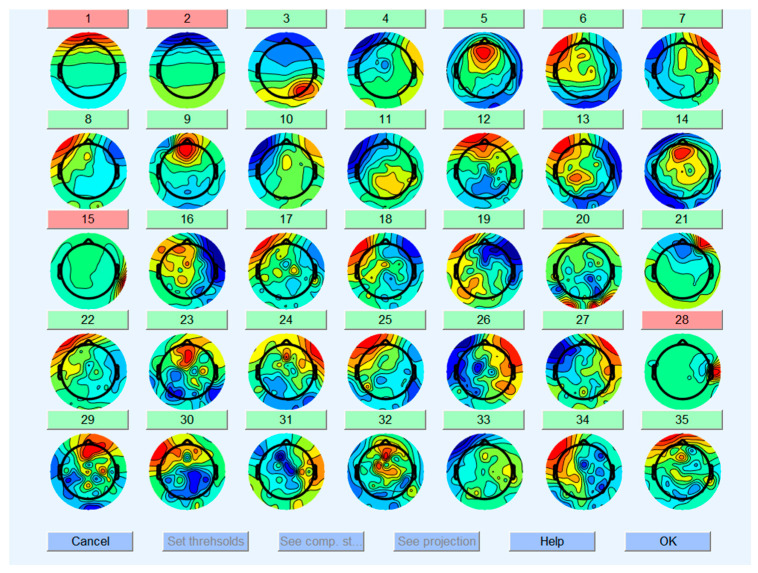
Removal of non-electroencephalogram components. Only the first 35 components are shown in the figure. The components marked in red have been removed.

Finally, an independent component analysis was performed on the data. Then, EEGLAB was used to draw the component map, and the electroophthalmic, electromyographic, electrocardiographic, and other clutter components were manually removed, as shown in Figure 5.

Afterwards, we analyzed the ERP in the hope of identifying differences between the real stimulus group and the sham stimulus group. We selected 10 channels, which were more centrally located in each brain region, including Fp1, Fp2, F3, F4, C3, C4, P3, P4, O1, and O2, as shown in Figure 4. First, we calculated the average ERP before and after stimulation for each group and compared the differences in N170 and P300 between the real stimulus group and the sham stimulus group. Then, we conducted a statistical analysis on the N170 and P300 of brain regions that exhibited significant differences. Due to the significant variations in brain activity among individuals, we did not directly utilize the ERP amplitude as the test parameter. Instead, we employed the relative index of X = (amplitude after stimulation/amplitude before stimulation) for analysis.

(1) $$X=\frac{\left|Peak\;or\;trough\;values\;after\;stimulation\right|}{\left|Peak\;or\;trough\;values\;before\;stimulation\right|}$$

We recorded the N170 and P300 before and after stimulation for each subject and calculated the (amplitude after stimulation/amplitude before stimulation) for these two indices. We used the rank-sum test in non-parametric statistics to compare the real stimulus group and the sham stimulus group and obtained the relevant results.

Theoretically, after the cerebellar target received TMS, observable changes should occur in the whole brain network. We used phase-locked values (PLV) to construct brain networks in different frequency bands, with the goal of identifying changes in brain networks before and after stimulation. First, we filtered the EEG signals according to the conventional frequency bands. Then, we used the task-related EEG signals to construct the PLV brain network. After obtaining the brain network, we used the top 30% of connection strengths as the threshold to generate a sparse matrix of the brain network. Then, we focused on studying the brain networks in the Theta (4–8 Hz), Alpha (9–12 Hz), and Beta (13–30 Hz) frequency bands. We utilized the BCT toolbox to compute the parameters associated with brain network efficiency and conducted a comparison. The global efficiency was compared within each group before and after stimulation, while the local efficiency was compared based on the division of brain regions. The test method was the rank-sum test.

## 3. Analysis and Results

### 3.1. ERP Results

**Figure 6:**
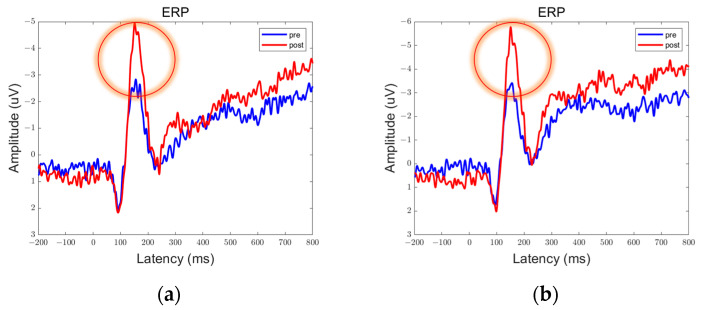
ERPs in the prefrontal lobe in the Group 5 Hz. There were statistically significant differences in the data in the encircled circles. (**a**) Fp1; (**b**) Fp2.

**Figure 7:**
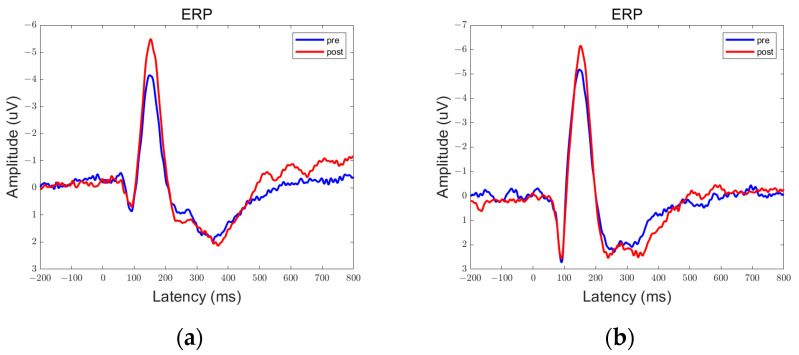
ERPs in the parietal lobe in the Group 5 Hz. (**a**) P3; (**b**) P4.

**Figure 8:**
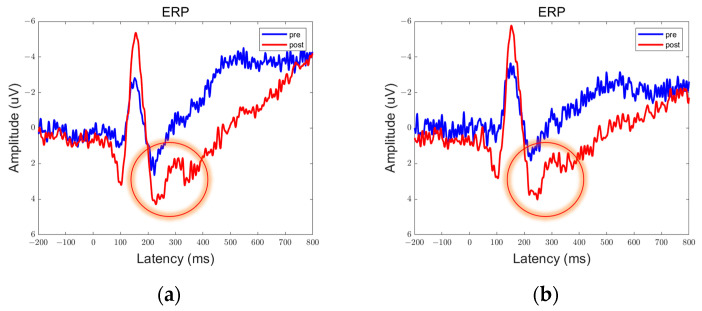
ERPs in the prefrontal lobe in the Group 20 Hz. There were statistically significant differences in the data in the encircled circles. (**a**) Fp1; (**b**) Fp2.

**Figure 9:**
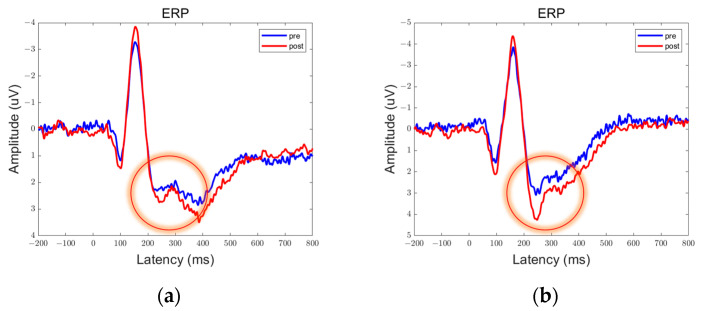
ERPs in the parietal lobe in the Group 20 Hz. There were statistically significant differences in the data in the encircled circles. (**a**) P3; (**b**) P4.

**Figure 10:**
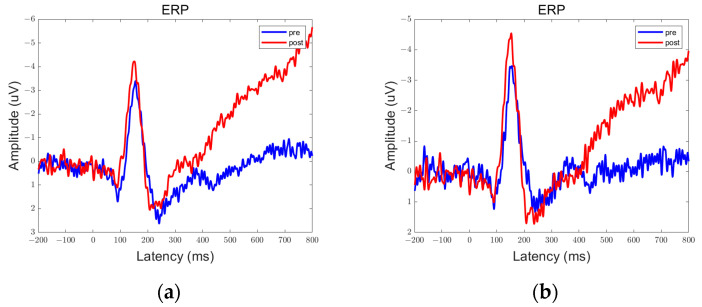
ERPs in the prefrontal lobe in the Group Sham. (**a**) Fp1; (**b**) Fp2.

**Figure 11:**
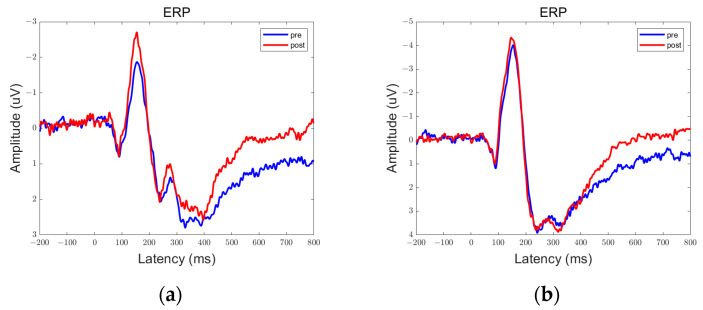
ERPs in the parietal lobe in the Group Sham. (**a**) P3; (**b**) P4.

In the data obtained from this experiment, there are clear peaks and troughs observed at approximately 100 ms, 170 ms, and 300 ms. Therefore, these points of interest will be the focus of our analysis. Among them, the prefrontal and parietal lobes showed significant differences between pre- and post-stimulation contrasts and sham stimulation contrasts, as shown in Figure 6, Figure 7, Figure 8, Figure 9, Figure 10 and Figure 11.

We defined the trough between 150 ms and 200 ms in the ERP as N170 and the peak between 250 ms and 350 ms as P300. By comparison, we can see that both 5 Hz and 20 Hz stimulation have some effects on the ERPs. In the parietal lobe, both stimuli increased the amplitude of the N170, and the amplitude of the P300 was increased by the 20 Hz stimulus. In the prefrontal cortex, 5 Hz stimulation increased the amplitude of N170, while 20 Hz stimulation increased the amplitude of P300. Sham stimulation did not cause any significant changes.

We averaged the ERP of Fp1 and Fp2 in the prefrontal lobe to obtain the average ERP of the left and right prefrontal lobes. Similarly, we averaged the ERP of P3 and P4 in the parietal lobe to obtain the average ERP of the left and right parietal lobes. We manually extracted the P300 and N170 for each subject to prepare for statistical analysis.

The test statistic X is shown in Table 2 and Table 3, and we obtained the p-values using the rank-sum test as shown in the tables.

It can be observed that stimulation at 5 Hz resulted in alterations in the N170 component of the prefrontal lobe, while stimulation at 20 Hz led to changes in the P300 component of the prefrontal lobe. In addition, the p-value between the 20 Hz stimulation group and the sham stimulation group in the P300 of the parietal lobe was 0.058, which is very close to the 0.05 threshold. Therefore, we can tentatively assume that the 20 Hz stimulation has caused changes in the P300 of the parietal lobe, although the changes are not very significant. All of these changes represent absolute increases in amplitude because, for the components with significant changes, the mean value of the true stimulus was larger than that of the sham stimulus.

### 3.2. Brain Network Results

The metrics we observe are global efficiency and local efficiency. After 5 Hz and 20 Hz stimulation, the global efficiency significantly improved, while sham stimulation showed no significant change. After 5 Hz stimulation, the local efficiency of the central area significantly decreased, while 20 Hz stimulation and sham stimulation showed no significant change. The changes brought about by 5 Hz were concentrated in the Theta band, and there was no significant change in the Alpha and Beta bands. However, the changes brought about by 20 Hz were concentrated in the Alpha band, and there was no significant change in the Theta and Beta bands.

First, is the global efficiency contrast in the Theta band, as shown in Table 4.

It can be seen that the 5 Hz stimulation significantly improved the global efficiency of the Theta band.

Then, the same method was used to analyze the global efficiency of the Alpha band and Beta band. No significant difference was found before and after stimulation in the Beta band. However, in the Alpha band, a significant increase in global efficiency was found after 20 Hz stimulation, while no difference was observed in the other groups. The results of the Alpha band are shown in Table 5.

**Figure 12:**
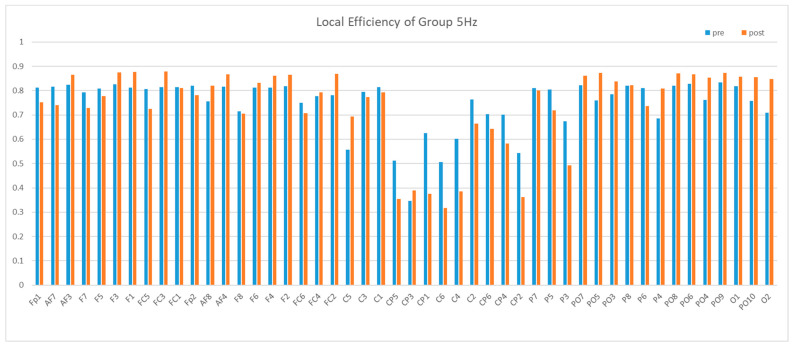
Mean local efficiency of Group 5 Hz in the Theta band. We can see a reduction between pre and post in CP5-CP2.

**Figure 13:**
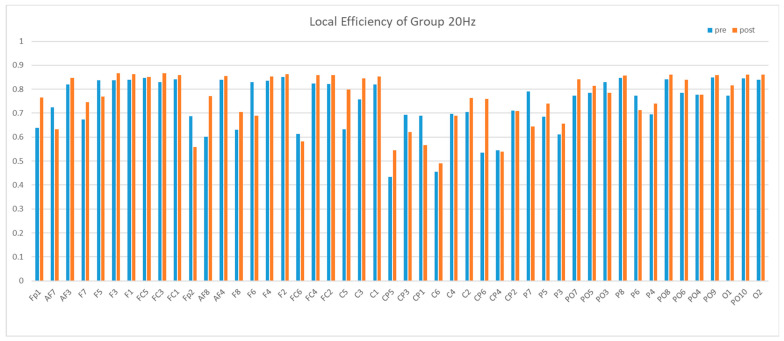
Mean local efficiency of Group 20 Hz in the Theta band.

**Figure 14:**
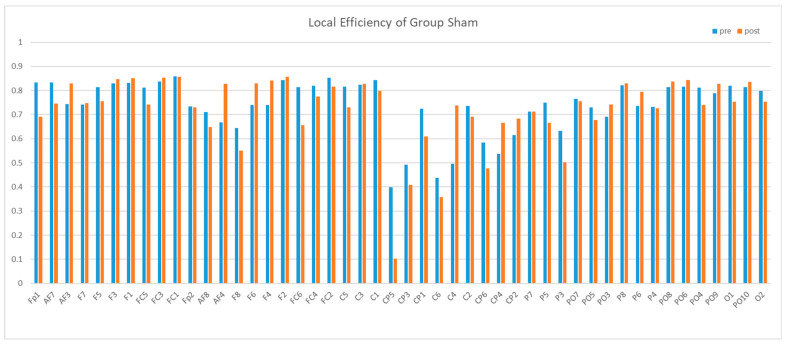
Mean local efficiency of Group Sham in the Theta band.

Next, we compared the local efficiency. The local efficiency of the central region was significantly lower than that of other brain regions, and this difference was not influenced by rTMS. Then, after 5 Hz stimulation, there was a significant additional decrease in local efficiency of the central region, whereas 20 Hz stimulation and false stimulation did not produce this effect, as shown in Figure 12, Figure 13 and Figure 14.

In order to obtain a statistically significant conclusion, the local efficiency before and after the stimulation of each subject in the 5 Hz group was analyzed separately. The average local efficiency of the central region (C1-C6, CP1-CP6) was used as an indicator to calculate a value for each subject before and after stimulation, as shown in Table 6.

It can be observed that there is a significant difference before and after 5 Hz stimulation. The local efficiency of the central area was significantly lower after stimulation compared to before stimulation.

In terms of the local efficiency of the Alpha and Beta bands, we did not observe any significant changes before and after stimulation.

## 4. Discussion

P300 is considered to be an electrophysiological indicator of working memory. It is generally believed that its amplitude is positively correlated with the allocation, updating, and execution processes of working memory resources. Some studies also suggest that its amplitude is related to the difficulty of working memory tasks [40,41]. N170 is a component elicited by words or face images. It is commonly believed that this component only reflects the behavior of the participants “seeing” the images. However, some studies also suggest that the amplitude and latency of this component are influenced by past memories [42]. The 5 Hz stimulation increased N170 amplitude in the prefrontal lobes, while 20 Hz increased P300 amplitude in the prefrontal and parietal lobes. The results showed that 5 Hz stimulation promoted the brain activity of seeing characters more, while 20 Hz stimulation promoted thinking. Collectively, both cerebellar target stimulations enhanced ERP responses in the 2-back task relative to the sham-stimulation group, demonstrating the effectiveness of rTMS of the cerebellar target on a working memory task.

The results of brain network analysis showed that the sham stimulation had no significant effect on the local and global efficiency of the brain network. The 5 Hz stimulation increased the global efficiency in the Theta band and decreased the local efficiency in the central region, and the 20 Hz stimulation increased the global efficiency in the Alpha band. In either group, the local efficiency in the central region was lower than that in the other brain regions. We believe that this is due to the fact that the function of the central region is not much related to working memory, and this brain region has low participation in the 2-back task, so there is low local efficiency. The decrease in local efficiency of 5 Hz stimulation in the central area means that the brain resources are better allocated, which means the brain resources are more efficient to participate in the task. The effect of 5 Hz and 20 Hz stimulation on global efficiency is reflected in different frequency bands. We suspect that this is due to a certain resonance effect between the frequency of rTMS and the EEG frequency, but the specific relationship and mechanism of action may need further research to explain.

Our research aims to find the stimulation parameters suitable for improving working memory through rTMS. It is known that TMS has been widely used to treat various diseases, such as Parkinson’s disease, stroke, neuralgia, depression, and schizophrenia [43]. Some studies have reported that TMS can improve cognitive abilities in patients with schizophrenia, but there is a lack of evidence from evidence-based medicine [44]. For cognitive impairments such as Alzheimer’s disease, single-target brain interventions are not highly effective, and there are differences in the evaluation of effects, which limits the application of interventions targeting a single area of the brain [45]. In summary, further evidence is needed to determine the efficacy of classical brain target stimulation for diseases with impaired cognitive function. In recent years, there have been increasing studies on TMS of the cerebellum. In a study on subjects applying theta-burst stimulation (TBS) to the cerebellar Crus I, researchers evaluated the behavioral performance of completing a contextual task before and after stimulation. It was found that cerebellar theta stimulation improved the encoding of contextual memory [46]. Mark A et al. [21] found that intermittent theta-burst stimulation (iTBS) of the lateral Crus I/II of the human cerebellum enhances the connectivity of the default mode network in the cerebral cortex. Our study also found that 5 Hz and 20 Hz rTMS can enhance ERP activity and improve the working memory of subjects. Although John E. Desmond et al. [47] conducted a Sternberg working memory task on 17 healthy subjects, where single-pulse TMS stimulation was immediately given after letter cues, the results showed a significant increase in reaction time after TMS stimulation. Although John E et al. also intervened in the cerebellum, our TMS protocol was completely different from theirs. First, we did not provide stimulation during the task state. Second, we used rTMS. Additionally, our study focuses on changes in ERP and brain networks, which sets it apart from their study. We believe that different TMS protocols have distinct effects.

Two limitations of this study are that the subjects were exclusively healthy young adults and that the behavioral data before and after stimulation were not analyzed in a systematic manner. Therefore, this conclusion cannot be directly applied to the treatment of cognitive and memory impairment-related diseases. However, it does offer new ideas for early screening and prevention of such diseases. For example, rTMS may be used to delay the decline of cognitive and memory function in healthy individuals during the aging process. Of course, this needs to be further studied, such as including a group of elderly individuals without any pre-existing conditions who are susceptible to disease. In addition, we also collected behavioral data simultaneously during the experiment, which will be included in the scope of subsequent data analysis.

## 5. Conclusions

In conclusion, from the perspective of EEG, rTMS targeting the cerebellar Crus II subregion can improve the performance of the 2-back task. The cerebellum does participate in or regulate the execution of the 2-back task. The 5 Hz stimulation and 20 Hz stimulation have obvious effects, but the effects are different.

## Figures and Tables

**Table 1:** Subjects and their basic information.

5 Hz			20 Hz			Sham		
No.	Gender	Age	No.	Gender	Age	No.	Gender	Age
1	female	24	1	male	24	1	female	24
2	female	27	2	female	24	2	female	23
3	female	28	3	male	23	3	female	27
4	male	23	4	female	24	4	male	27
5	female	24	5	male	27	5	male	26
6	female	24	6	male	23	6	female	24
7	female	26	7	male	25	7	male	24
8	female	24	8	female	24	8	female	24
9	female	24	9	female	25	9	female	26
10	female	24	10	male	25	10	female	24
11	female	25	11	male	23			
12	male	25	12	male	25			
13	male	27						
14	female	24						

**Table 2:** ERP analysis of the prefrontal lobe.

	Number of Subjects *	1	2	3	4	5	6	7	8	9	10	11	12
$\frac{N170\;post}{N170\;pre}$	Group 5 Hz	1.660	3.789	0.719	1.807	2.475	1.792	1.923	1.400	2.078	1.671	1.201	4.948
	Group 20 Hz	1.928	2.412	1.350	0.640	0.477	2.161	0.820	0.843	0.394	1.219		
	Group Sham	0.481	1.685	0.650	1.329	1.007	0.907	0.999	1.173				
	P (5 Hz and Sham)	0.005											
	P (20 Hz and Sham)	0.897											
$\frac{P300\;post}{P300\;pre}$	Group 5 Hz	2.521	0.989	1.320	0.112	6.361	1.009	0.224	2.871	0.670	0.347	2.613	
	Group 20 Hz	2.699	1.429	1.464	1.351	1.481	2.055	9.102	2.243	2.128	1.801		
	Group Sham	0.615	1.475	0.243	1.110	0.566	1.102	0.735	1.350				
	P (5 Hz and Sham)	0.657											
	P (20 Hz and Sham)	0.0003											

* Some subjects whose ERP was not evident have been eliminated.

**Table 3:** ERP analysis of the parietal lobe.

	Number of Subjects *	1	2	3	4	5	6	7	8	9	10	11
$\frac{N170\;post}{N170\;pre}$	Group 5 Hz	0.949	1.158	1.533	1.551	0.969	1.189	1.568	0.811	1.170	1.077	1.473
	Group 20 Hz	1.073	1.104	0.895	1.175	1.640	1.026	1.153	1.128	1.382	1.053	0.930
	Group Sham	0.727	1.246	0.836	1.005	1.474	0.950	1.042	1.454	1.085		
	P (5 Hz and Sham)	0.288										
	P (20 Hz and Sham)	0.543										
$\frac{P300\;post}{P300\;pre}$	Group 5 Hz	1.158	1.071	1.145	1.155	2.084	1.833	1.009	1.057	1.216	1.172	0.677
	Group 20 Hz	1.354	1.535	2.733	1.189	1.004	1.222	0.973	3.457	1.316	1.213	1.011
	Group Sham	0.898	0.881	1.475	0.905	0.605	0.918	1.104	2.271	1.192		
	P (5 Hz and Sham)	0.323										
	P (20 Hz and Sham)	0.058										

* Some subjects whose ERP was not evident have been eliminated.

**Table 4:** Global efficiency comparison in the Theta band.

No.	Group 5 Hz		Group 20 Hz		Group Sham	
	Pre	Post	Pre	Post	Pre	Post
1	0.594	0.619	0.480	0.539	0.713	0.605
2	0.498	0.600	0.699	0.500	0.549	0.521
3	0.538	0.503	0.505	0.623	0.509	0.478
4	0.552	0.649	0.544	0.507	0.478	0.484
5	0.487	0.490	0.552	0.526	0.573	0.636
6	0.573	0.683	0.617	0.640	0.527	0.757
7	0.492	0.518	0.556	0.532	0.504	0.512
8	0.508	0.506	0.477	0.516	0.458	0.503
9	0.482	0.612	0.520	0.507	0.508	0.505
10	0.495	0.481	0.487	0.738	0.469	0.471
11	0.504	0.576	0.552	0.551		
12	0.496	0.661	0.475	0.470		
13	0.478	0.545				
14	0.566	0.510				
	Ave = 0.519	Ave = 0.568	Ave = 0.646	Ave = 0.665	Ave = 0.529	Ave = 0.547
	P = 0.0366		P = 0.1939		P = 0.7337	

**Table 5:** Global efficiency comparison in the Alpha band.

No.	Group 5 Hz		Group 20 Hz		Group Sham	
	Pre	Post	Pre	Post	Pre	Post
1	0.574	0.660	0.498	0.596	0.663	0.587
2	0.488	0.498	0.518	0.625	0.506	0.522
3	0.611	0.553	0.473	0.535	0.622	0.537
4	0.608	0.672	0.569	0.586	0.516	0.478
5	0.539	0.538	0.530	0.702	0.576	0.573
6	0.684	0.535	0.514	0.540	0.577	0.527
7	0.620	0.716	0.537	0.508	0.603	0.582
8	0.599	0.581	0.478	0.507	0.486	0.546
9	0.605	0.618	0.541	0.559	0.554	0.540
10	0.620	0.538	0.519	0.635	0.626	0.627
11	0.468	0.498	0.500	0.584		
12	0.566	0.605	0.545	0.506		
13	0.486	0.551				
14	0.511	0.509				
	Ave = 0.570	Ave = 0.577	Ave = 0.622	Ave = 0.688	Ave = 0.573	Ave = 0.552
	P = 0.9817		P = 0.0226		P = 0.4727	

**Table 6:** Mean local efficiency of central region of Group 5 Hz.

No.	1	2	3	4	5	6	7	8	9	10	11	12	13	14
**pre**	0.767	0.699	0.643	0.588	0.559	0.517	0.678	0.638	0.669	0.629	0.731	0.565	0.686	0.558
**post**	0.457	0.424	0.462	0.375	0.611	0.391	0.682	0.507	0.516	0.601	0.557	0.149	0.696	0.749
										**sum**	**pre**	8.715	**P = 0.0159**	
											**post**	7.388		

## Data Availability

The data are available upon reasonable request.
